# *tert*-Butyl 6-amino-3,4-dihydro-2*H*-1,4-benzoxazine-4-carboxyl­ate

**DOI:** 10.1107/S160053681004777X

**Published:** 2010-11-24

**Authors:** Xiao-Bo Gu, Meng-Jun Jiang, Gang-Ming Cai, Yao-Yuan Zhou

**Affiliations:** a Key Laboratory of Nuclear Medicine, Ministry of Health, Jiangsu Key Laboratory of Molecular Nuclear Medicine, Jiangsu Institute of Nuclear Medicine, Wuxi 214063, People’s Republic of China

## Abstract

The title mol­ecule, C_13_H_18_N_2_O_3_, contains a benzene ring fused to an oxazine ring and one *tert*-but­oxy­carbonyl group bound to the N atom of the oxazine ring. A weak intra­molecular C—H⋯O inter­action occurs. In the crystal, inter­molecular N—H⋯O and C—H⋯O hydrogen bonds stack the mol­ecules down the *b* axis. Weak C—H⋯N contacts connect the stacks, generating a three-dimensional network.

## Related literature

For the pharmacological properties of phenyl­morpholine derivatives, see: Albanese *et al.* (2003 ▶); La *et al.* (2008 ▶); McCormick *et al.* (2008 ▶). For related structures, see: Chen *et al.* (2003 ▶); Olmstead *et al.* (2003 ▶); Vergeer *et al.* (1999 ▶).
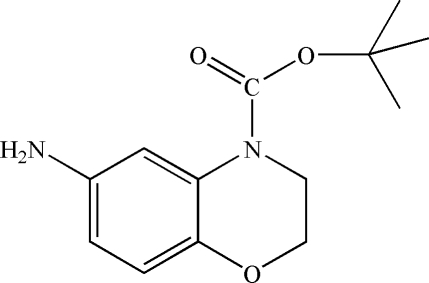


## Experimental

### 

#### Crystal data

C_13_H_18_N_2_O_3_
*M* *_r_* = 250.29
Monoclinic, 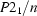

*a* = 9.439 (4) Å
*b* = 7.941 (3) Å
*c* = 17.598 (7) Å
β = 97.235 (6)°
*V* = 1308.6 (8) Å^3^
*Z* = 4
Mo *K*α radiation
μ = 0.09 mm^−1^
*T* = 103 K
0.37 × 0.27 × 0.21 mm

#### Data collection

Rigaku AFC10/Saturn724+ diffractometer
13574 measured reflections
3816 independent reflections
3118 reflections with *I* > 2σ(*I*)
*R*_int_ = 0.035

#### Refinement

*R*[*F* ^2^ > 2σ(*F* ^2^)] = 0.047
*wR*(*F* ^2^) = 0.106
*S* = 1.00
3816 reflections
174 parameters
H atoms treated by a mixture of independent and constrained refinement
Δρ_max_ = 0.41 e Å^−3^
Δρ_min_ = −0.19 e Å^−3^

### 

Data collection: *CrystalClear* (Rigaku, 2008 ▶); cell refinement: *CrystalClear*; data reduction: *CrystalClear*; program(s) used to solve structure: *SHELXS97* (Sheldrick, 2008 ▶); program(s) used to refine structure: *SHELXL97* (Sheldrick, 2008 ▶); molecular graphics: *SHELXTL* (Sheldrick, 2008 ▶); software used to prepare material for publication: *SHELXTL*.

## Supplementary Material

Crystal structure: contains datablocks I, global. DOI: 10.1107/S160053681004777X/sj5053sup1.cif

Structure factors: contains datablocks I. DOI: 10.1107/S160053681004777X/sj5053Isup2.hkl

Additional supplementary materials:  crystallographic information; 3D view; checkCIF report

## Figures and Tables

**Table 1:** Hydrogen-bond geometry (Å, °)

*D*—H⋯*A*	*D*—H	H⋯*A*	*D*⋯*A*	*D*—H⋯*A*
N2—H2*B*⋯O3^i^	0.885 (17)	2.088 (17)	2.9581 (19)	167.4 (16)
C2—H2⋯O3	0.95	2.23	2.7981 (18)	117
C7—H7*A*⋯O1^ii^	0.99	2.55	3.364 (2)	139
C13—H13*B*⋯N2	0.98	2.61	3.586 (2)	172

Symmetry codes: (i) 
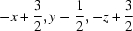
; (ii) 
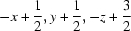
.

**Fig. 1.**
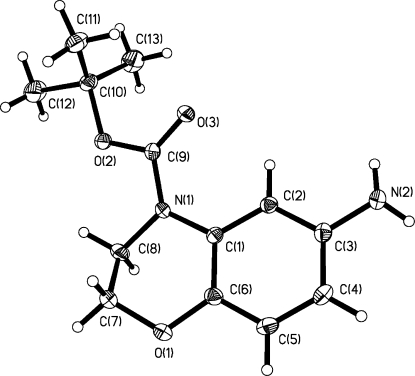
A view of the title compound with the atomic numbering scheme with displacement ellipsoids drawn at the 50% probability level.

## supplementary crystallographic information

### Comment

The title compound, (I), is an important phenylmorpholine derivative. Phenylmorpholine compounds are used as α2 C adrenergic receptor agonists. (McCormick *et al.*, 2008). Numerous phenylmorpholine derivatives possess various other pharmacological properties. (Albanese, *et al.*, 2003; La, *et al.*, 2008).

We report here the crystal structure of the title compound. (Fig. 1). The title molecule of (I) contains a benzene ring fused to an oxazine ring and one *tert*-butoxycarbonyl bound to the N atom. The N1—C1 bond distance is 1.4230 (14)Å and agrees with literature values (Vergeer, *et al.*, 1999; Chen, *et al.*, 2003; Olmstead, *et al.*, 2003). The six-membered heterocyclic ring adopts a half-chair conformation with atoms C7 and C8 lying out of the plane through the remaining four atoms by 0.3264 (14) and -0.4174 (13) Å, respectively.

In the crystal structure intermolecular N2—H2B···O3 and C7—H7···O1 hydrogen bonds stack the molecules down the *b* axis.  Weak C13—H13B···N2 contacts connect the stacks generating a three-dimensional network, Table 1.

### Experimental

The title compound was crystallized from a mixed solvent composed of dichloromethane and hexane (1:1); colorless block-shaped crystals were obtained after several days.

### Refinement

Positional parameters of all the H atoms bonded to C atoms were calculated geometrically and were allowed to ride on the C atoms to which they were bonded, with C—H distances of 0.95Å (CH), 0.98Å (CH_3_) or 0.99Å (CH_2_), and with Uiso(H) =1.2 or 1.5 (methyl) Ueq of the parent atoms. The H-atoms bound to N were found in a difference map and allowed to refine freely.

### Crystal data

**Table:**

C_13_H_18_N_2_O_3_	*F*(000) = 536
*M**_r_* = 250.29	*D*_x_ = 1.270 Mg m^−^^3^
Monoclinic, *P*2_1_/*n*	Mo *K*α radiation, λ = 0.71073 Å
Hall symbol: -P 2yn	Cell parameters from 3844 reflections
*a* = 9.439 (4) Å	θ = 3.6–30.0°
*b* = 7.941 (3) Å	µ = 0.09 mm^−^^1^
*c* = 17.598 (7) Å	*T* = 103 K
β = 97.235 (6)°	Block, colorless
*V* = 1308.6 (8) Å^3^	0.37 × 0.27 × 0.21 mm
*Z* = 4	

### Data collection

**Table:**

Rigaku AFC10/Saturn724+ diffractometer	3118 reflections with *I* > 2σ(*I*)
Radiation source: Rotating Anode	*R*_int_ = 0.035
graphite	θ_max_ = 30.0°, θ_min_ = 2.3°
Detector resolution: 28.5714 pixels mm^-1^	*h* = −13→13
φ and ω scans	*k* = −11→11
13574 measured reflections	*l* = −22→24
3816 independent reflections	

### Refinement

**Table:**

Refinement on *F*^2^	Primary atom site location: structure-invariant direct methods
Least-squares matrix: full	Secondary atom site location: difference Fourier map
*R*[*F*^2^ > 2σ(*F*^2^)] = 0.047	Hydrogen site location: inferred from neighbouring sites
*wR*(*F*^2^) = 0.106	H atoms treated by a mixture of independent and constrained refinement
*S* = 1.00	*w* = 1/[σ^2^(*F*_o_^2^) + (0.0431*P*)^2^ + 0.506*P*] where *P* = (*F*_o_^2^ + 2*F*_c_^2^)/3
3816 reflections	(Δ/σ)_max_ = 0.001
174 parameters	Δρ_max_ = 0.41 e Å^−^^3^
0 restraints	Δρ_min_ = −0.19 e Å^−^^3^

### Special details

**Table:**

Geometry. All e.s.d.'s (except the e.s.d. in the dihedral angle between two l.s. planes) are estimated using the full covariance matrix. The cell e.s.d.'s are taken into account individually in the estimation of e.s.d.'s in distances, angles and torsion angles; correlations between e.s.d.'s in cell parameters are only used when they are defined by crystal symmetry. An approximate (isotropic) treatment of cell e.s.d.'s is used for estimating e.s.d.'s involving l.s. planes.
Refinement. Refinement of *F*^2^ against ALL reflections. The weighted *R*-factor *wR* and goodness of fit *S* are based on *F*^2^, conventional *R*-factors *R* are based on *F*, with *F* set to zero for negative *F*^2^. The threshold expression of *F*^2^ > σ(*F*^2^) is used only for calculating *R*-factors(gt) *etc*. and is not relevant to the choice of reflections for refinement. *R*-factors based on *F*^2^ are statistically about twice as large as those based on *F*, and *R*- factors based on ALL data will be even larger.

### Fractional atomic coordinates and isotropic or equivalent isotropic displacement parameters (Å^2^)

**Table:**

	*x*	*y*	*z*	*U*_iso_*/*U*_eq_	
O1	0.29075 (9)	0.23440 (12)	0.77441 (5)	0.0238 (2)	
O2	0.27308 (9)	0.60469 (11)	0.55727 (5)	0.01769 (18)	
O3	0.47393 (9)	0.70065 (11)	0.62807 (5)	0.01852 (19)	
N1	0.36964 (10)	0.45345 (12)	0.65823 (6)	0.0148 (2)	
N2	0.84770 (11)	0.48161 (15)	0.80355 (7)	0.0226 (2)	
C1	0.46953 (11)	0.41260 (14)	0.72306 (6)	0.0138 (2)	
C2	0.61108 (12)	0.46877 (14)	0.73224 (6)	0.0153 (2)	
H2	0.6415	0.5440	0.6956	0.018*	
C3	0.70861 (12)	0.41698 (15)	0.79396 (7)	0.0166 (2)	
C4	0.66399 (13)	0.30549 (15)	0.84764 (7)	0.0191 (2)	
H4	0.7294	0.2684	0.8898	0.023*	
C5	0.52391 (13)	0.24943 (15)	0.83905 (7)	0.0200 (2)	
H5	0.4941	0.1738	0.8757	0.024*	
C6	0.42614 (12)	0.30153 (15)	0.77789 (7)	0.0172 (2)	
C7	0.18660 (13)	0.31174 (17)	0.71949 (8)	0.0243 (3)	
H7A	0.1574	0.4212	0.7395	0.029*	
H7B	0.1010	0.2389	0.7105	0.029*	
C8	0.24682 (13)	0.33916 (16)	0.64505 (7)	0.0220 (3)	
H8A	0.2771	0.2301	0.6251	0.026*	
H8B	0.1728	0.3884	0.6066	0.026*	
C9	0.37957 (11)	0.59648 (14)	0.61556 (6)	0.0140 (2)	
C10	0.27444 (13)	0.74056 (16)	0.49941 (7)	0.0195 (2)	
C11	0.25934 (15)	0.91226 (17)	0.53576 (8)	0.0268 (3)	
H11A	0.1790	0.9102	0.5660	0.040*	
H11B	0.2419	0.9978	0.4955	0.040*	
H11C	0.3474	0.9395	0.5692	0.040*	
C12	0.14095 (16)	0.7002 (2)	0.44419 (8)	0.0338 (3)	
H12A	0.1490	0.5864	0.4236	0.051*	
H12B	0.1314	0.7817	0.4020	0.051*	
H12C	0.0567	0.7065	0.4714	0.051*	
C13	0.40675 (16)	0.7250 (2)	0.45926 (8)	0.0305 (3)	
H13A	0.4910	0.7576	0.4946	0.046*	
H13B	0.3977	0.7992	0.4144	0.046*	
H13C	0.4171	0.6082	0.4429	0.046*	
H2A	0.8731 (17)	0.538 (2)	0.7639 (10)	0.030 (4)*	
H2B	0.9124 (19)	0.408 (2)	0.8234 (10)	0.039 (5)*	

### Atomic displacement parameters (Å^2^)

**Table:**

	*U*^11^	*U*^22^	*U*^33^	*U*^12^	*U*^13^	*U*^23^
O1	0.0165 (4)	0.0260 (5)	0.0295 (5)	−0.0026 (3)	0.0048 (3)	0.0108 (4)
O2	0.0174 (4)	0.0176 (4)	0.0170 (4)	−0.0026 (3)	−0.0021 (3)	0.0026 (3)
O3	0.0171 (4)	0.0165 (4)	0.0210 (4)	−0.0041 (3)	−0.0015 (3)	0.0022 (3)
N1	0.0124 (4)	0.0144 (5)	0.0174 (5)	−0.0023 (3)	0.0006 (3)	0.0006 (4)
N2	0.0170 (5)	0.0221 (6)	0.0268 (6)	−0.0006 (4)	−0.0040 (4)	0.0055 (5)
C1	0.0147 (5)	0.0122 (5)	0.0146 (5)	0.0021 (4)	0.0023 (4)	−0.0006 (4)
C2	0.0159 (5)	0.0137 (5)	0.0164 (5)	0.0012 (4)	0.0029 (4)	0.0006 (4)
C3	0.0164 (5)	0.0145 (5)	0.0184 (5)	0.0022 (4)	0.0007 (4)	−0.0026 (4)
C4	0.0220 (6)	0.0182 (6)	0.0167 (5)	0.0054 (4)	0.0002 (4)	0.0012 (4)
C5	0.0243 (6)	0.0180 (6)	0.0185 (6)	0.0027 (5)	0.0057 (4)	0.0045 (5)
C6	0.0161 (5)	0.0162 (5)	0.0201 (5)	0.0009 (4)	0.0057 (4)	0.0012 (4)
C7	0.0151 (5)	0.0241 (7)	0.0337 (7)	−0.0021 (5)	0.0025 (5)	0.0094 (5)
C8	0.0191 (6)	0.0200 (6)	0.0257 (6)	−0.0085 (5)	−0.0015 (5)	0.0034 (5)
C9	0.0136 (5)	0.0147 (5)	0.0138 (5)	0.0014 (4)	0.0022 (4)	−0.0015 (4)
C10	0.0216 (6)	0.0199 (6)	0.0160 (5)	−0.0003 (5)	−0.0017 (4)	0.0041 (4)
C11	0.0323 (7)	0.0191 (6)	0.0280 (7)	0.0036 (5)	−0.0006 (5)	0.0032 (5)
C12	0.0350 (8)	0.0345 (8)	0.0274 (7)	−0.0065 (6)	−0.0143 (6)	0.0071 (6)
C13	0.0328 (7)	0.0392 (8)	0.0209 (6)	0.0035 (6)	0.0090 (5)	0.0066 (6)

### Geometric parameters (Å, °)

**Table:**

O1—C6	1.3789 (15)	C5—H5	0.9500
O1—C7	1.4278 (15)	C7—C8	1.5081 (19)
O2—C9	1.3443 (13)	C7—H7A	0.9900
O2—C10	1.4848 (15)	C7—H7B	0.9900
O3—C9	1.2152 (14)	C8—H8A	0.9900
N1—C9	1.3713 (15)	C8—H8B	0.9900
N1—C1	1.4230 (14)	C10—C13	1.5154 (19)
N1—C8	1.4675 (15)	C10—C11	1.5204 (19)
N2—C3	1.3999 (16)	C10—C12	1.5253 (17)
N2—H2A	0.887 (17)	C11—H11A	0.9800
N2—H2B	0.884 (19)	C11—H11B	0.9800
C1—C2	1.3985 (16)	C11—H11C	0.9800
C1—C6	1.4058 (16)	C12—H12A	0.9800
C2—C3	1.3945 (16)	C12—H12B	0.9800
C2—H2	0.9500	C12—H12C	0.9800
C3—C4	1.3977 (17)	C13—H13A	0.9800
C4—C5	1.3853 (18)	C13—H13B	0.9800
C4—H4	0.9500	C13—H13C	0.9800
C5—C6	1.3896 (17)		
			
C6—O1—C7	114.78 (10)	N1—C8—H8A	109.8
C9—O2—C10	119.23 (9)	C7—C8—H8A	109.8
C9—N1—C1	122.94 (9)	N1—C8—H8B	109.8
C9—N1—C8	122.19 (10)	C7—C8—H8B	109.8
C1—N1—C8	114.60 (9)	H8A—C8—H8B	108.3
C3—N2—H2A	115.6 (10)	O3—C9—O2	124.46 (10)
C3—N2—H2B	113.3 (12)	O3—C9—N1	124.31 (10)
H2A—N2—H2B	113.7 (16)	O2—C9—N1	111.22 (9)
C2—C1—C6	118.52 (10)	O2—C10—C13	109.84 (10)
C2—C1—N1	123.15 (10)	O2—C10—C11	110.72 (10)
C6—C1—N1	118.19 (10)	C13—C10—C11	113.31 (12)
C3—C2—C1	121.55 (11)	O2—C10—C12	101.90 (10)
C3—C2—H2	119.2	C13—C10—C12	110.43 (12)
C1—C2—H2	119.2	C11—C10—C12	110.06 (11)
C2—C3—C4	119.19 (11)	C10—C11—H11A	109.5
C2—C3—N2	120.19 (11)	C10—C11—H11B	109.5
C4—C3—N2	120.57 (11)	H11A—C11—H11B	109.5
C5—C4—C3	119.64 (11)	C10—C11—H11C	109.5
C5—C4—H4	120.2	H11A—C11—H11C	109.5
C3—C4—H4	120.2	H11B—C11—H11C	109.5
C4—C5—C6	121.37 (11)	C10—C12—H12A	109.5
C4—C5—H5	119.3	C10—C12—H12B	109.5
C6—C5—H5	119.3	H12A—C12—H12B	109.5
O1—C6—C5	116.15 (11)	C10—C12—H12C	109.5
O1—C6—C1	124.09 (10)	H12A—C12—H12C	109.5
C5—C6—C1	119.73 (11)	H12B—C12—H12C	109.5
O1—C7—C8	110.33 (10)	C10—C13—H13A	109.5
O1—C7—H7A	109.6	C10—C13—H13B	109.5
C8—C7—H7A	109.6	H13A—C13—H13B	109.5
O1—C7—H7B	109.6	C10—C13—H13C	109.5
C8—C7—H7B	109.6	H13A—C13—H13C	109.5
H7A—C7—H7B	108.1	H13B—C13—H13C	109.5
N1—C8—C7	109.19 (10)		
			
C9—N1—C1—C2	−26.57 (17)	N1—C1—C6—O1	−2.62 (17)
C8—N1—C1—C2	159.29 (11)	C2—C1—C6—C5	−0.48 (17)
C9—N1—C1—C6	157.74 (11)	N1—C1—C6—C5	175.41 (11)
C8—N1—C1—C6	−16.40 (15)	C6—O1—C7—C8	44.10 (15)
C6—C1—C2—C3	0.09 (17)	C9—N1—C8—C7	−126.86 (12)
N1—C1—C2—C3	−175.59 (10)	C1—N1—C8—C7	47.33 (14)
C1—C2—C3—C4	0.40 (17)	O1—C7—C8—N1	−61.83 (14)
C1—C2—C3—N2	−176.97 (11)	C10—O2—C9—O3	5.54 (17)
C2—C3—C4—C5	−0.49 (17)	C10—O2—C9—N1	−173.22 (9)
N2—C3—C4—C5	176.87 (11)	C1—N1—C9—O3	0.71 (17)
C3—C4—C5—C6	0.10 (18)	C8—N1—C9—O3	174.42 (11)
C7—O1—C6—C5	169.42 (11)	C1—N1—C9—O2	179.48 (10)
C7—O1—C6—C1	−12.48 (17)	C8—N1—C9—O2	−6.81 (15)
C4—C5—C6—O1	178.58 (11)	C9—O2—C10—C13	61.25 (14)
C4—C5—C6—C1	0.40 (18)	C9—O2—C10—C11	−64.64 (14)
C2—C1—C6—O1	−178.52 (11)	C9—O2—C10—C12	178.33 (11)

### Hydrogen-bond geometry (Å, °)

**Table:**

*D*—H···*A*	*D*—H	H···*A*	*D*···*A*	*D*—H···*A*
N2—H2B···O3^i^	0.885 (17)	2.088 (17)	2.9581 (19)	167.4 (16)
C2—H2···O3	0.95	2.23	2.7981 (18)	117
C7—H7A···O1^ii^	0.99	2.55	3.364 (2)	139
C13—H13B···N2^iii^	0.98	2.61	3.586 (2)	172

Symmetry codes: (i) −*x*+3/2, *y*−1/2, −*z*+3/2; (ii) −*x*+1/2, *y*+1/2, −*z*+3/2; (iii) .
